# Purification and Characterization of β-Mannanase Derived from *Rhizopus microsporus* var. *rhizopodiformis* Expressed in *Komagataella phaffii*

**DOI:** 10.3390/foods13203324

**Published:** 2024-10-19

**Authors:** Jinghua Qu, Jie Long, Xingfei Li, Xing Zhou, Long Chen, Chao Qiu, Zhengyu Jin

**Affiliations:** 1 The State Key Laboratory of Food Science and Resources, Jiangnan University, 1800 Lihu Road, Wuxi 214122, China; 6210112035@stu.jiangnan.edu.cn (J.Q.); jielong@jiangnan.edu.cn (J.L.);; 2 School of Food Science and Technology, Jiangnan University, 1800 Lihu Road, Wuxi 214122, China; 3 Collaborative Innovation Center of Food Safety and Quality Control in Jiangsu Province, Jiangnan University, Wuxi 214122, China

**Keywords:** reduced endogenous secretory proteins, hydrolysis properties, high temperature, *Pichia pastoris*

## Abstract

The demand for food-grade β-mannanases, ideal for high-temperature baking, is increasing. Using the *Komagataella phaffii* (*P. pastoris*) expression system for β-mannanase production, this study aimed to enhance purification methods. We evaluated better conditions for production and purification of β-mannanase (*PpRm*Man134A) from recombinant *P. pastoris* X-33, focusing on a higher purity and reducing the production of endogenous secretory proteins in fermentation. By adjusting carbon and nitrogen sources, culture time, and temperature, we controlled cell growth to reduce the production of endogenous secretory proteins. The better-evaluated conditions involved culturing recombinant *P. pastoris* in 70 mL buffered glycerol complex medium for 24 h at 30 °C, then in modified buffered methanol-complex medium with 0.91% (*w*/*v*) methanol, 0.56% (*w*/*v*) sorbitol, and 0.48% (*w*/*v*) mannitol for another 24 h, which improved the *PpRm*Man134A yield and reduced endogenous secretory proteins, shortening the fermentation time by 72 h. An affordable purification method using ultrafiltration and salt-out precipitation was utilized. *PpRm*Man134A showed thermostability up to 100 °C and effectively degraded locust bean gum into smaller fragments, mainly producing mannotriose. In conclusion, with its enhanced purity due to reduced levels of endogenous secretory proteins, purified *PpRm*Man134A emerges as a promising enzyme for high-temperature baking applications.

## 1. Introduction

Adding soybean residue to white bread increases its fiber and protein content but negatively affects volume, density, and texture [1]. The application of hemicellulase can mitigate these issues [2]. Specifically, β-mannanase (endo-1,4-β-D-mannanases, EC 3.2.1.78) plays a pivotal role in breaking down the predominant hemicellulose in soybeans, mannan, into mannooligosaccharides (MOSs) [3]. This degradation of the cell wall structure of soybean cells [4] not only improves nutrient utilization and texture but also results in MOS, which are commonly used as prebiotics [5,6]. The rising demand for food-grade β-mannanases for use in high-temperature baking processes underscores the necessity of effective and pure enzyme production.

The methylotrophic yeast *Komagataella phaffii* (*P. pastoris*) is a well-established system for the production of biopharmaceuticals and industrial enzymes [7], including food-grade β-mannanases [8,9,10]. Despite the considerable CO_2_ emissions arising from large-scale fermentation of industrial yeast, these CO_2_ emissions can be repurposed in the industrial methanol production process [11], contributing to carbon neutrality. The limited production of endogenous secretory proteins (ESPs) by *P. pastoris* facilitates the purification of recombinant proteins (RPs) [12]. Moreover, *P. pastoris* grows more rapidly and expresses higher-yield RPs than *Escherichia coli* (*E. coli*) and *Saccharomyces cerevisiae* (*S. cerevisiae*) expression systems [13,14]. However, the focus of current research has predominantly been on enhancing the production of RPs [10,13,15], with less attention given to the purification of these proteins. The expression of RPs in *P. pastoris* under the alcohol oxidase promoter is associated with the production of ESPs in the culture supernatant [16]. These ESPs necessitate efficient removal strategies such as chromatography, which is complex and costly [7,17], or the currently used membrane-based approaches [17].

In light of the energy crisis and the pursuit of carbon neutrality, cheaper methods to express and purify β-mannanases are needed. This study was specifically aimed at reducing the presence of ESPs in the fermentation supernatant, thereby enhancing the purity of the β-mannanase product. However, ESPs are linked to cell growth [18]. Thus, controlling cell growth during methanol induction is crucial. Characterizing and optimizing a strain’s traits and process variables often requires significant time and money [19]. Various methods and mathematical tools are used to achieve a high biomass and RP yield [20], but many focus on linear equations or ordinary differential equations [20], where increased protein production correlates with cellular growth.

Most research has focused on the link between energy metabolism and cell growth, overlooking the connections between metabolism, growth, and reproduction [15,20]. A new theory has been developed and tested to understand these relationships, concluding that metabolism, growth, and reproduction are inextricably linked [21]:

*E_T_* = *E_M_* + *E_G_* + *E_R_*, (1)

where *E_T_*, total energy intake; *E_M_*, energy to self-maintenance; *E_G_*, energy to cell growth; *E_R_*, energy to reproduction.

Optimal growth and reproduction within a finite lifespan result in allometric metabolic scaling [21], including in fungi [22]. Evolution has fine-tuned these processes to produce intraspecific metabolic allometries [21]. To ensure *P. pastoris* increases its wet cell weight (WCW) during enzyme production and minimizes ESPs from cell death [23], refer to Equation (2) expanded from Equation (1) for biological growth:

(2) $$dm/dt=\left[fa_{E}M^{b_{E_{T}}}-\left(fa_{E}M^{b_{E_{T}}}/M^{b_{E_{R}}}\right)m^{b_{E_{R}}}\right]/C_{m}$$

where *m*, organism mass; *dm*/*dt*, the change in *m* over time (*t*); *a_E_*, the scaling coefficient; *b_E_*, the scaling exponent (including *E_T_* and *E_R_*); *M*, maximum mass before reproductive maturity; *f*, production is allocated a fraction (*f*) of *E_T_* by organisms; *C_m_*, used to convert energy units into mass units during organism biosynthesis [21].

In recombinant *P. pastoris*, the secretory expression vector used the α-factor signals from *S. cerevisiae* to aid protein secretion [7]. Since α-factor production is linked to mating [24], the mating environment—including carbon and nitrogen sources, time, and temperature—can influence β-mannanase production. The temperature’s effect on WCW [23,25] is detailed in Equation (3) expanded from Equation (2):

(3) $$a_{E}{M^{b}}^{E_{T}}=B_{0}e^{-\frac{E}{kT}}m^{\frac{3}{4}},$$

where $a_{E}{M^{b}}^{E_{T}}$, the whole organism metabolic rate; *e*, the average activation energy of rate-limiting biochemical metabolic reactions; *k*, Boltzmann’s constant; *T*, absolute temperature; *B*_0_, a taxon-dependent and metabolic-state-dependent normalization constant; *m*, the mass of the organism [26,27].

In summary, this study aimed to better evaluate and control cell growth conditions (carbon source, nitrogen source, time, and temperature), based on Equations (1)–(3), to enhance the purity of β-mannanase produced by recombinant *P. pastoris*. Then, cost-effective purification methods, ultrafiltration, and salt-out precipitation were used for β-mannanase (*Rm*Man134A [28]), referred to as *PpRm*Man134A in this study. The enzymatic properties and hydrolyzed products of *PpRm*Man134A were also evaluated.

## 2. Materials and Methods

### 2.1. Materials and Strains

Yeast Nitrogen Base (YNB) without amino acids and the Yeast Genomic DNA Extraction Kit were acquired from Sorlabio (Beijing, China). Thermo Fisher Scientific (Shanghai, China) provided Yeast Extract Powder and Zeocin. Casein peptone and peptone were acquired from Macklin (Shanghai, China). Coomassie Blue Super Fast Staining Solution, restriction endonuclease *Sac* I, *P. pastoris* X-33 Yeast Glycerol Stock, and *E. coli* TOP10 Super Competent Cells were purchased from Beyotime Biotech (Shanghai, China). Food-grade xanthan gum (XG), guar gum (GB), locust bean gum (LBG), konjac glucomannan (KGM), and glucomannan (GM) were obtained from Henan Wanbang Chemical Technology Co., Ltd. (Zhengzhou, China). Chemical reagents that are not separately identified as coming from other companies in this study were purchased from Sinopharm Chemical Reagents (Shanghai, China).

### 2.2. Construction and Mutants

The gene of *Rm*Man134A (accession number MF538624 in the NCBI GenBank database) was optimized by *P. pastoris* condon preference. Both forward and reverse primers contained *Xho* I [29] and *Not* I sites. KEX2 cleavage site [29] was located ahead of the mature *Rm*Man134A gene. The terminator (TAA) was located ahead of *Not* I (Figure S1). This final inserted gene was chemically synthesized and cloned into *Xho* I-*Not* I sites of pPICZαA (Azenta, Suzhou, China), yielding the plasmid pPICZαA-*RmMan134A*. The recombinant *E. coli* TOP10 was constructed following instructions from TOP10 Super Competent Cells (Beyotime, Shanghai, China), which was confirmed by DNA sequencing performed by Azenta (Suzhou, China).

To integrate genes into the chromosomal DNA of *P. pastoris* at the AOX1 locus, the recombinant plasmids were linearized by *Sac* I. The transformation into *P. pastoris* X-33, which produced the highest yield of RP among the four *P. pastoris* strains [30], was accomplished by electroporation based on the instructions in [7]. The transformants with large colonies were selected to extract genomic DNA. They were confirmed by DNA sequencing performed by Azenta (Suzhou, China).

### 2.3. Protein Expression

The recombinant *P. pastoris* was precultured for 24 h at 30 °C in a rotary shaking incubator (ZQZY-784AV, Shanghai Zhchu Instrument Co., Ltd., Shanghai, China) using buffered minimal glycerol complex (BMGY) medium (pH 6.37, 100 mM potassium phosphate buffer). Cells were then centrifuged (TGL-18M, Shanghai Luxiangyi centrifuge Instrument Co., Ltd., Shanghai, China) at 5000× *g* for 10 min at 25 °C, and resuspended in 100 mL modified buffered methanol-complex (MBMMY) medium with 0.5% (*w*/*v*) Tween 20 [31] and 100 mM potassium phosphate buffer (pH 6.37) to induce expression. The cells were cultured in BMGY and MBMMY media, both of which contained 1.34% (*w*/*v*) YNB, at 200 rpm. The conditions for enzyme production were adjusted according to Tables S1–S4, derived from the optimal conditions for enzyme production in *P. pastoris* [13,32,33] and Equations (1)–(3).

Yeast cells were then harvested at 5000× *g* for 10 min at 25 °C to measure WCW, followed by collecting the fermentation supernatant.

### 2.4. Protein Purification

The fermentation supernatant (10 mL) from methods B1, B5 (Table S2), and D9 (Table S4) was centrifuged in a 3 kDa ultrafiltration tube at 5000× *g* for 40 min at 25 °C. Among them, the key differences among schemes B1, B5, and D9 were in the initial medium composition, additive concentrations, and fermentation conditions. B1 and B5 varied in initial casein peptone levels and supplement volumes, whereas D9 used higher initial concentrations of casein peptone and yeast extract and a shorter fermentation time and maintained a constant temperature. Then, ammonium sulfate (5–10%, *w*/*v*) was added at 25 °C until the concentrated liquid’s turbidity stabilized. The solution was divided into 2 mL centrifuge tubes and centrifuged (Sorvall Legend Micro 21R, Thermo Fisher Scientific, Osterode am Harz, Germany) at 10,000× *g* for 5 min at 4 °C. The solution and precipitate were separated, with the precipitate dissolved in ddH_2_O, centrifuged (TGL-18M, Shanghai Luxiangyi centrifuge Instrument Co., Ltd., Shanghai, China), and concentrated multiple times using a 3 kDa ultrafiltration tube through 50 mM citrate buffer (pH 5.5).

### 2.5. Protein Analysis

The protein concentration was measured using an Enhanced BCA Protein Assay Kit (Beyotime, Shanghai, China). SDS-PAGE was performed with a Tris-glycine gel (20% separating, 4% concentrating).

The molecular mass of *PpRm*Man134A was determined via gel filtration chromatography on a pre-equilibrated BioCore SEC-300 column (5 μm, 7.8 × 300 mm, Nano spectrum Analysis Technology (Suzhou) Co., Ltd., Suzhou, China) using 100 mM sodium phosphate buffer (pH 7.0) as the mobile phase. A Waters e2695 system (Waters, Milford, MA, USA) equipped with a 2489 UV/Vis Detector was used at 220 nm. The protein was eluted at a flow rate of 0.5 mL/min at 30 °C. Calibration was used with 69385 Protein Standard Mix 15–600 kDa for testing of SEC/GFC columns (Sigma, Shanghai, China).

### 2.6. Determination of Enzyme Properties

The activity of *PpRm*Man134A was assessed using the 3,5-dinitrosalicylic acid (DNS) method [28,34], employing 0.5% (*w*/*v*) LBG as the substrate. The DNS solution consisted of 1.6% (*w*/*v*) sodium hydroxide (NaOH), 1% (*w*/*v*) DNS, and 30% (*w*/*v*) potassium sodium tartrate. We preheated 1 mL substrate, which was dissolved in 50 mM sodium citrate buffer (pH 5.5) to 40 °C using a WB100-2 water bath (JOANLAB, Huzhou, China). Then, we added 0.1 mL diluted fermentation supernatant to the preheated substrate and thoroughly mixed the contents. The reaction mixtures were incubated (JULABO SW22, Ulebo Technology (Beijing) Co., Ltd., Beijing, China) at 40 °C for 5 min. Following the addition of 1.5 mL DNS, the reaction was boiled (FQ-501, Mieal, Zhongshan, China) for 5 min to terminate it. Enzyme activity in the reaction mixture was promptly measured at 540 nm (Spectrophotometer1510, Thermo Fisher Scientific, Vantaa, Finland) after dilution to 10 mL.

Sodium citrate and citric acid are commonly employed as food additives [35,36], while hydrochloric acid and sodium hydroxide are used for pH adjustment in food. Therefore, a 50 mM citric acid–sodium citrate buffer system with a pH range of 2.0–10.0 was utilized in this study.

Buffers (pH 3.0–8.0) were prepared by combining 50 mM citric acid and 50 mM sodium citrate solutions in varying volumes. To achieve a pH of 2.0, an appropriate amount of hydrochloric acid was added to the 50 mM citric acid. Solutions (pH 9.0, 10.0) were prepared by adding NaOH to the 50 mM sodium citrate solution. The optimal pH for *PpRm*Man134A was determined by conducting experiments in 50 mM of each buffer at a temperature of 37 °C using a WB100-2 water bath.

After incubating *PpRm*Man134A at 37 °C (WB100-2, JOANLAB, Huzhou, China) for 30 min in each buffer, the remaining activity of the enzyme was assessed. The optimal temperature range of the enzyme in 50 mM (pH 5.5) citrate buffer was determined to be 20 °C–80 °C.

The thermostability of *PpRm*Man134A was tested by heating (HCJ-6E, Changzhou Enyi Instrument Co., Ltd., Changzhou, China) it at 40 °C, 50 °C, and 60 °C for 360 min; at 70 °C for 130 min; at 80 °C and 90 °C for 120 min; and at 100 °C for 4 min. Additionally, it was measured at 25 °C for 5 min after the 100 °C treatment for 4 min.

*PpRm*Man134A was incubated with 20 mM of various-ion solution and 20 mM of other chemicals [ethylenediaminetetra-acetic acid (EDTA), urea, methanol, alcohol] for 30 min at 37 °C. In 50 mM (pH 5.5) citrate buffer at 37 °C, residual enzyme activities were determined by the standard assay. Kinetic parameters were calculated for 5 min at 40 °C and pH 5.5 using different concentrations of LBG (1–9 mg/mL).

Additionally, the substrate specificity of *PpRm*Man134A towards 5 mg/mL food-grade xanthan gum (XG), guar gum (GB), locust bean gum (LBG), konjac glucomannan (KGM), and glucomannan (GM) was determined using the DNS method under conditions that maximize enzyme activity.

### 2.7. Hydrolysis Properties

The hydrolysis of 0.2 mg *PpRm*Man134A toward 10 mg/mL XG, GB, LBG, konjac KGM, and GM at 40 °C for 20 h was analyzed by thin-layer chromatography (TLC) [28]. The hydrolysates were then boiled (FQ-501, Mieal, Zhongshan, China) for 20 min and centrifuged (Sorvall Legend Micro 21R, Thermo Fisher Scientific, Osterode am Harz, Germany) at 10,000× *g* for 7 min at 4 °C. The clarified supernatant was centrifuged using 100 kDa and 3 kDa ultrafiltration tubes (Cobetter, Hangzhou, China) at 3000× *g* for 15 min at 20 °C to obtain oligosaccharide products.

The molecular weight was measured in the solution retained by the 3 kDa ultrafiltration tube. The hydrolysis products of GB and LBG were analyzed by high-performance gel filtration chromatography (HPGFC, Waters 1525, Waters, Milford, MA, USA) with a 2410 RI Detector and Empower 3.0 Workstation on a pre-equilibrated Ultrahydrogel^TM^ Linear column (7.8 × 300 mm, Waters, Milford, MA, USA) with 100 mM sodium nitrate. The product was eluted at 0.5 mL/min at 40 °C. Calibration standards, including Dextran T-2000 (2000 kDa), Dextran T-300 (300.6 kDa), Dextran T-150 (135,030 Da), Dextran T-100 (70,000 Da), Dextran T-10 (9750 Da), Dextran T-5 (2700 Da), and glucose (180 Da), were obtained from the China Food and Drug Administration.

The oligosaccharide filtrate from a 3 kDa ultrafiltration tube was analyzed using high-performance liquid chromatography (HPLC, Waters e2695, Waters, Milford, MA, USA) with a 2414 RI Detector and Empower 3.0 Workstation on an APS-2 Hypersil^TM^ column (250 × 4.6 mm, Thermo Fisher Scientific, Shanghai, China) pre-equilibrated with 75/25 (*v*/*v*) acetonitrile/water and eluted at 1 mL/min at 30 °C.

The standards (Meryer, Suzhou, China) in TCL and HPGFC included D-glucose (G1), sucrose (G2), maltotriose (G3), maltotetraose (G4), maltopentaose (G5), maltohexaose (G6), D-mannose (M1), and mannotriose (M3). The molecular weight and oligosaccharide content of LBG products hydrolyzed by *PpRm*Man134A for 5 min, 30 min, 60 min, and 120 min were measured similarly.

### 2.8. Analytical Methods

Triplicate experiments were conducted independently. The data were processed as mean values and standard deviation (SD). Excel 2024 (Microsoft Office 365) was used to perform a *t* test (two-tailed *p-*value).

## 3. Results and Discussion

### 3.1. Better-Evaluated Conditions for Enzymatic Production of PpRmMan134A

The content of ESPs in fermentation supernatant rises with the cell number [18]. Based on Equations (2) and (3), if the culture temperature is fixed, reducing the content of ESPs requires controlling *m* and *M* in the supernatant, which is challenging, but the energy input to the medium can be managed. Additionally, during the decay phase (*dm*/*dt* < 0), cell death releases ESPs, further increasing their content in the supernatant. Equation (2) indicates that cell growth (*dm*/*dt*) can be controlled by adjusting the energy input into the medium. To sum up, energy regulation can modulate the energy input by adjusting the quantities of nitrogen and carbon sources added in MBMMY medium, thereby controlling cell growth. Different nitrogen sources can initially be added to the medium, but Equations (2) and (3) show that daily addition of sufficient carbon sources is necessary for cell growth and RP production.

As cell numbers increase, so does carbon demand. RP production peaked at 2–2.5% (*w*/*v*) methanol, with a minimum of 0.5% (*w*/*v*) needed [13]. Excessive methanol raises ESPs in the fermentation supernatant. Thus, when examining sorbitol and mannitol’s effects on *PpRm*Man134A production, 0.59–0.79% (*w*/*v*) methanol was added daily (Table S1). Sorbitol and mannitol also boost enzyme activity and RP production [13,32,33,37]. Previous research found that sorbitol/methanol ratios of 2:5, 1:4, and 1:2 (*w*/*w*) were optimal for producing RPs in a shake flask culture with an inorganic nitrogen medium [13]. However, in MBMMY medium, which includes both organic and inorganic nitrogen sources and mannitol, excessive sorbitol/methanol was unnecessary. The 1:10 (*w*/*w*) sorbitol/methanol ratio was chosen because the amino acids and nucleic acids in organic nitrogen sources can also supply energy [32]. Gu et al. found that a 1:20 (*w*/*w*) mannitol/methanol ratio was ideal for RP production, and a 1:10 (*w*/*w*) ratio was best for *P. pastoris* growth [33]. Consequently, this study used a 1:9 (*w*/*w*) sorbitol and mannitol/methanol mix, with a 5:4 (*w*/*w*) sorbitol/mannitol ratio.

Notably, higher cell numbers lead to increased ESPs [18], necessitating control of cell numbers in the fermentation solution. To manage cell numbers during fermentation, methanol, sorbitol, and mannitol should be reduced within 72 h–120 h according to Equation (2). Consequently, in schemes A5–A9, methanol, sorbitol, and mannitol levels were increased within 24 h–72 h and decreased within 72 h–120 h. In contrast, methanol levels in schemes A1–A4 remained constant as a control (Table S1).

**Figure 1:**
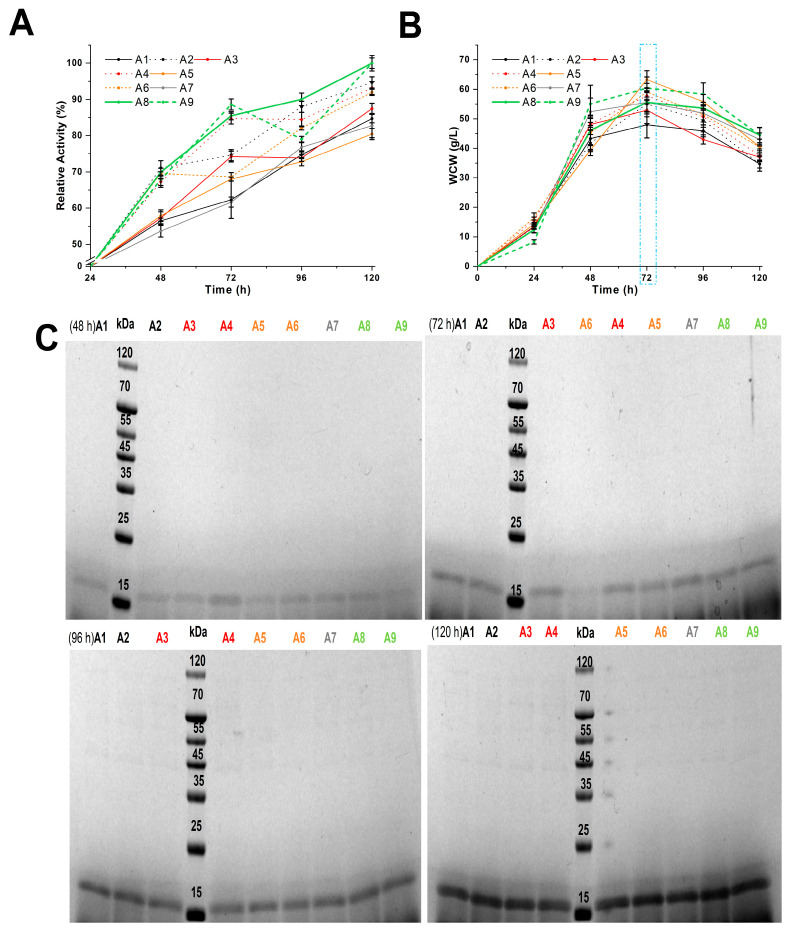
Better-evaluated conditions for enzymatic production of schemes A1–A9. Within 120 h of fermentation, the relative enzyme activity (**A**) and wet cell weight (**B**) were changed. The maximum enzyme activity in the fermentation supernatant according to scheme A8 at 120 h was defined to be 100%, where the calculated enzyme amount was 0.35 mg. (**C**) SDS-PAGE analysis of *PpRm*Man134A in the supernatants.

Schemes A1–A9 (Table S1) investigated the effects of peptone, casein peptone, and various carbon sources on *PpRm*Man134A production at 30 °C, evaluating enzyme activity, WCW, and ESPs in the fermentation supernatant (Figure 1). The final fermentation supernatant of schemes A8 and A9 had the highest enzyme activity cultured according to schemes A1–A9 (Figure 1A). Scheme A9 included 1 g more yeast extract than scheme A8, which replaced 2% (*w*/*v*) casein peptone with 2% (*w*/*v*) peptone (Table S1), making scheme A8 more advantageous. Figure 1A indicated higher enzyme activity in the fermentation broth when casein peptone was used as the nitrogen source in MBMMY medium, likely due to its higher tyrosine content than peptone [38], which was conducive to the production of *PpRm*Man134A (18.2 kDa). So, the impact of varying the casein peptone and yeast extract quality on the enzyme activity of *PpRm*Man134A was tested, as shown in schemes B1–B5 (Table S2).

In schemes A1–A9, all recombinant *P. pastoris* entered the decay phase (*dm*/*dt* < 0) within 72 h to 120 h of fermentation (Figure 1B), releasing ESPs (42 kDa–110 kDa) as they died (Figure 1C). Schemes A5–A9, which included sorbitol and mannitol, produced fewer ESPs than schemes A1–A4, which used only methanol (Figure 1C). This is because sorbitol and mannitol enhance ATP production via the tricarboxylic acid cycle, reducing the reliance on alcohol oxidase in recombinant *P. pastoris* [13].

Studies indicated that lowering the culture temperature from 30 °C to 20 °C boosted RP yield, although *P. pastoris* grew best at 28 °C–30 °C [13]. Based on Equations (1)–(3), it is evident that while *P. pastoris’*s energy metabolism slows at lower temperatures, more energy is directed towards reproduction and enzyme production, but additional energy is required for survival. Lower culture temperatures and increased carbon sources raise RP production costs. Hence, in schemes B1–B5, the culture temperature of *P. Pastoris* in MBMMY medium was adjusted to 30 °C, 29 °C, 28 °C, and 27.2 °C every 24 h (Table S2).

**Figure 2:**
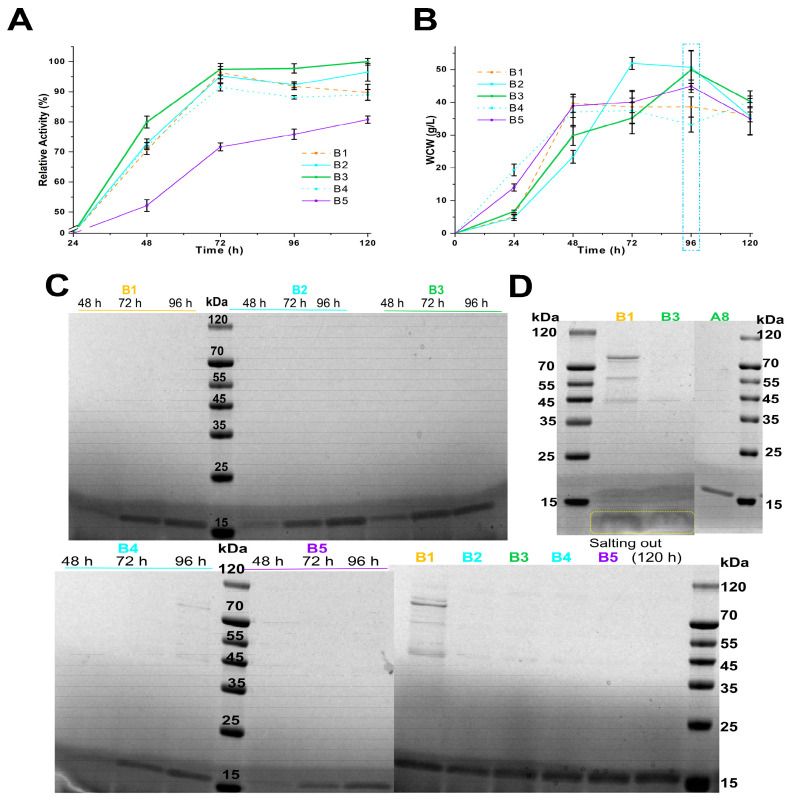
Better-evaluated conditions for enzymatic production of schemes B1-B5. The change in the relative enzyme activity (**A**) and wet cell weight (**B**) within 120 h of fermentation. The maximum enzyme activity in the fermentation supernatant according to scheme B3 at 120 h was defined to be 100%, where the calculated enzyme amount was 0.35 mg. (**C**) SDS-PAGE analysis of *PpRm*Man134A in the supernatants. (**D**) SDS-PAGE analysis of *PpRm*Man134A after salting out. Proteins smaller than 15 kDa were framed in yellow.

Scheme B3, which used 100 mL MBMMY medium with 2% (*w*/*v*) casein peptone and 1% (*w*/*v*) yeast extract, was the most effective for producing *PpRm*Man134A with the highest enzyme activity among schemes B1–B5, outperforming B4 and B5 (Figure 2A). The combination of sorbitol and mannitol was more beneficial for *PpRm*Man134A production than using sorbitol alone, as evidenced by schemes B2 and B3 (Figure 2A, Table S2). The decline phase (*dm*/*dt* < 0) for B1–B5 occurred within 96 h–120 h of fermentation, suggesting that reducing the temperature can slow cell metabolism and ESP release (Figure 2B).

Although *PpRm*Man134A produced in schemes A8, B1, and B3 was concentrated by three salt-out precipitations, schemes A8 and B3 had significantly fewer ESPs than scheme B1 (Figure 2D). An unknown protein under 15 kDa, possibly casein (5.8 kDa) derived from casein peptone [39], suggests casein peptone should not be used as a nitrogen source in MBMMY medium; peptone is preferable (Figure 2D). Additionally, schemes A8 and B3 showed 85.50% and 97.50% increases in relative enzyme activity within 24 h–72 h of fermentation, with no significant ESPs in the supernatant (Figure 1A, Figure 1C, Figure 2A and Figure 2C, respectively). However, during 48 h–72 h, their enzyme activity only increased by 15.54% and 17.57%, compared to 69.96% and 79.92% during 24 h–48 h (Figure 1A and Figure 2A). This was due to increased WCW, which led cells to allocate more energy to maintenance rather than growth and enzyme production, as explained by Equations (1)–(3). To mitigate energy inefficiency associated with prolonged fermentation, the fermentation time in schemes C and D was adjusted to 0 h–48 h.

However, this shorter duration significantly reduced the *PpRm*Man134A yield (Figure 1C and Figure 2C). According to Equation (2), a larger *M* directs energy towards reproduction and enzyme production instead of cell growth. Thus, to boost the *PpRm*Man134A yield, more *P. pastoris* should be transferred from BMGY to MBMMY medium, effectively increasing the total energy in BMGY medium. Consequently, in schemes C1–C10 and D1–D16, the BMGY medium volume was raised from 50 mL to 70 mL and more glycerol was added (Tables S3 and S4). Besides examining the impact of peptone and casein peptone on *PpRm*Man134A production in BMGY medium, it is also important to consider the peptone amount in MBMMY medium, the type and amount of carbon source, and the final potassium phosphate buffer concentration. Thus, schemes C1–C10 (Table S3) were created. Additionally, increasing the methanol concentration can boost the PR yield, with 2% (*w*/*v*) methanol being a commonly used concentration in several experiments [13]. So, 0.91% (*w*/*v*) methanol was selected.

In schemes C1–C10, where casein peptone was used as the nitrogen source in BMGY medium, 2% (*w*/*v*) peptone in MBMMY medium, sorbitol and mannitol as carbon sources, and 100 mM potassium phosphate buffer, scheme C4 showed the highest relative enzyme activity (Figure 3A). Although the concentrations of *PpRm*Man134A in schemes C1–C10 were higher than those in schemes A1–A9 and B1–B5 during 24 h–48 h of fermentation, the content of ESPs in fermentation supernatant also increased (Figure 1C, Figure 2C, and Figure 3B). This may be due to the high methanol concentration in the fermentation solution, which increased alcohol oxidase levels. Additionally, the relative enzyme activity was significantly lower (72.09% and 73.59%) when 0.56% (*w*/*v*) sorbitol was added to MBMMY medium compared to when extra 0.48% (*w*/*v*) mannitol was added (87.46–100%, Figure 3A).

In schemes D1–D16, the impact of varying peptone and methanol contents on *PpRm*Man134A production was examined, keeping sorbitol, mannitol and potassium phosphate buffer constant (Table S4). Methanol levels of 1.38–2% (*w*/*v*) resulted in higher ESPs than 0.91–1.38% (*w*/*v*) (schemes D1–D16, Figure 3C). Scheme D9, which used 100 mL MBMMY medium with 2% (*w*/*v*) peptone and 0.91% (*w*/*v*) methanol, was demonstrated to be the most effective for producing *PpRm*Man134A with the highest enzyme activity among schemes D1–D16 (Figure 3D).

**Figure 3:**
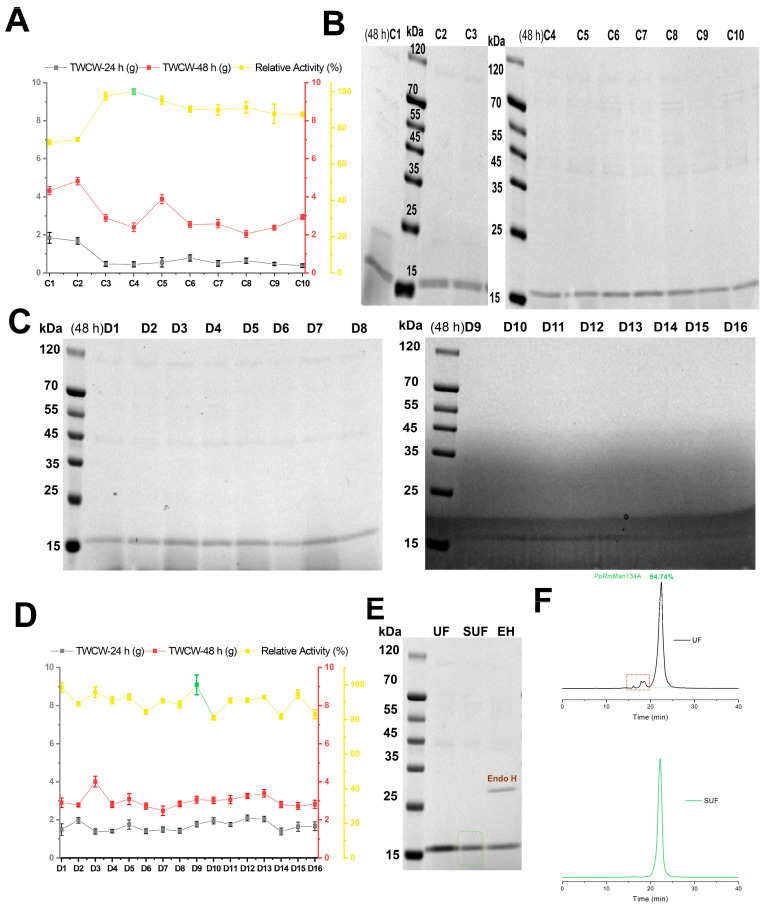
Better-evaluated conditions for enzymatic production of schemes C1–C10 and D1–D16. Schemes C1–C10 included relative enzyme activity and cell wet weight of *PpRm*Man134A (**A**), and SDS-PAGE analysis of *PpRm*Man134A in the supernatant (**B**). The maximum enzyme activity in the fermentation supernatant according to schemes C4 and D9 at 48 h was defined to be 100% and marked in green, where the calculated enzyme amount was 0.2 mg. Schemes D1–D16 included SDS-PAGE analysis of *PpRm*Man134A in the supernatant (**C**), relative enzyme activity, and cell wet weight (**D**). (**E**) SDS-PAGE analysis of purified *PpRm*Man134A is shown for ultrafiltration (UF), salting out and ultrafiltration (SUF), and Endo H deglycosylated *PpRm*Man134A (EH). (**F**) Molecular weight detection of *PpRm*Man134A after salting out and ultrafiltration was also included.

*PpRm*Man134A was produced under the conditions of scheme D9 (Table S4) and concentrated through ultrafiltration, salt-out precipitation, and another round of ultrafiltration. Protein molecular weight verification showed that 5.26% ESPs could be removed (Figure 3E,F). The purified *PpRm*Man134A, as shown in Figure 3F, was then used for further studies on enzymatic properties and hydrolysis capacity. Glycosylation validation using NetNGlyc 1.0 (https://services.healthtech.dtu.dk/services/NetNGlyc-1.0, accessed on 15 October 2023) and Endo H (Yeasen, Shanghai, China) revealed no N-glycosylation sites (Figure 3E).

In brief, using sorbitol, mannitol, and methanol as carbon sources in schemes A1–A9 to D1–D16 (Tables S1–S4) significantly reduced the ESP content in the fermentation supernatant (Figure 1C, Figure 2C, Figure 2D, Figure 3B and Figure 3C, respectively). The highest yield of *PpRm*Man134A was 3.56 mg/mL after 120 h in scheme A8, compared to 1.84 mg/mL after 48 h in scheme D9. Despite a lower yield, scheme D9 significantly reduced the fermentation time to 72 h, achieving a yield of 1.84 mg/mL *PpRm*Man134A per 24 h in MBMMY medium, much higher than scheme A8’s 0.89 mg/mL. Additionally, scheme D9 required fewer carbon sources and decreased the energy needed to maintain 30 °C for 72 h.

### 3.2. Enzymatic Properties of PpRmMan134A

Li et al. previously expressed recombinant *Rm*Man134A-Q17A (m*Rm*Man134A) in *Pichia pastoris* GS115 using a plasmid digested with *EcoR*I and *Not*I [34]. Unlike the method described in Section 2.2 of this study, their approach resulted in m*Rm*Man134A having extra amino acids at both the N-terminal and C-terminal of the enzyme (Figure S2). The target enzyme *Rm*Man134A has 162 active amino acids [28]. The additional 10 amino acids at both ends of m*Rm*Man134A, constituting 6.17% of the total, might explain the differing enzymatic properties between m*Rm*Man134A and *PpRm*Man134A.

**Figure 4:**
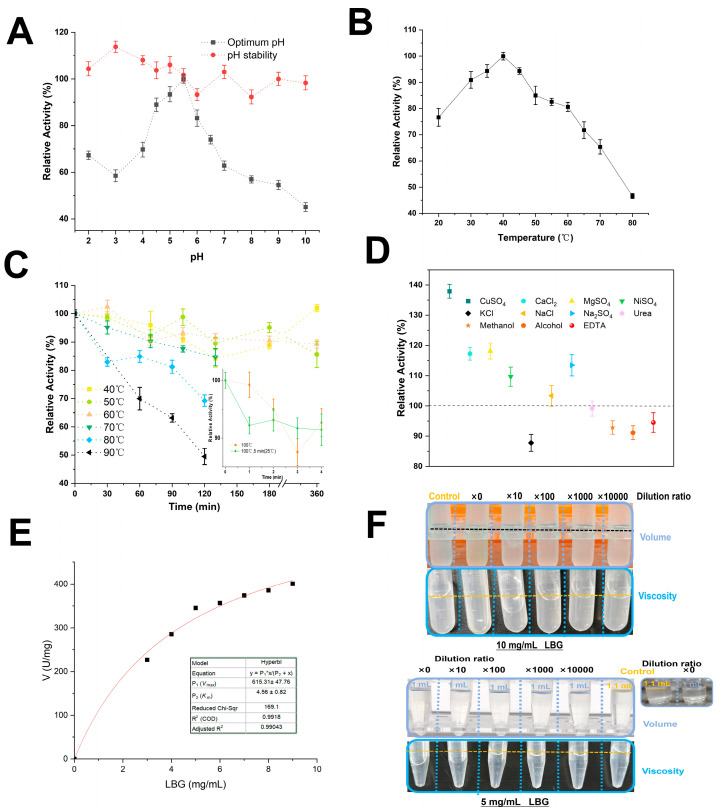
Enzymatic properties of *PpRm*Man134A: (**A**) optimum pH and stability, (**B**) optimum temperature, (**C**) thermostability, (**D**) impact of 20 mM of various chemical reagents, (**E**) kinetic parameters, and (**F**) volume and viscosity changes in the enzyme–substrate mixture. In (**F**), the reaction mixture comprised 0.1 mL enzyme solution at various dilutions (control: ddH_2_O) combined with 1 mL substrate solution (5 mg/mL or 10 mg/mL LBG) and was incubated at 40 °C for a duration of 5 min; LBG was dissolved in 50 mM sodium citrate buffer (pH 5.5); from ×0 to ×10,000: dilution ratio using ddH_2_O; ×0: undiluted 0.2 mg *PpRm*Man134A in 0.1 mL 50 mM sodium citrate buffer (pH 5.5).

Previous studies by You et al. showed that the Q17A mutation in *Rm*Man134A (expressed in *E. coli* Rosetta) did not alter its optimal pH and temperature [28]. Both *Rm*Man134A and m*Rm*Man134A had an optimal temperature of 50 °C and similar pH stability, with optimal pH values of 5.0 and 5.5 [28,34]. *PpRm*Man134A and m*Rm*Man134A [34] both showed peak activity at pH 5.5 and remained stable across a pH range of 4–10, retaining over 90% of their activity (Figure 4A).

However, the optimal temperature of *PpRm*Man134A was 40 °C (Figure 4B). *PpRm*Man134A without excess amino acid sequences at both ends showed better thermostability than m*Rm*Man134A, making it more suitable for the high temperature of grain baking [40]. *PpRm*Man134A demonstrated strong thermostability, retaining 80% of its initial enzyme activity at 40 °C–60 °C for 360 min and at 70 °C for 130 min. At 80 °C and 90 °C for 120 min, it still retained 69.26% and 49.5% residual activity, respectively. Over 85% of its initial activity was maintained after 4 min at 100 °C (Figure 4C). Additionally, *PpRm*Man134A was more stable after a preliminary 5 min treatment at 25 °C before high-temperature exposure (Figure 4C). In contrast, m*Rm*Man134A and *Rm*Man134A lost nearly all enzyme activity after 30 min at 65 °C–80 °C [28,34].

*PpRm*Man134A was activated by 20 mM Cu^2+^, Ca^2+^, Mg^2+^, Ni^2+^, and Na^+^, but inhibited by 20 mM K^+^, EDTA, alcohol, and methanol (Figure 4D). Its activity remained unaffected by 20 mM urea (Figure 4D). Notably, 10 mM SO4^2−^ had a stronger activation effect than 20 mM Cl^−^ at the same Na^+^ concentration (Figure 4D). Overall, most chemical reagents had minimal impacts, with the enzyme retaining over 85% activity after treatment. The *V*_max_ of *PpRm*Man134A was 615.31 U/mg of protein, with a *K*_m_ of 4.56 mg/mL (Figure 4E) for LBG. The *k*_cat_/*K*_m_ of *PpRm*Man134A on LBG was 1.13 mL/(mg·s). However, low concentrations (1 mg/mL, 2 mg/mL) of locust bean gum showed high error rates and were excluded from the fit.

Figure 4F indicates the viscosity and volume of LBG change when treated with *PpRm*Man134A at different dilutions. Although enzyme activity persisted after diluting 0.2 mg *PpRm*Man134A by 10,000 times and reacting it with LBG, the change in viscosity was minimal. The key change happened when undiluted 0.2 mg *PpRm*Man134A reacted with LBG. Therefore, a low dilution ratio was selected to facilitate the subsequent production of oligosaccharides.

### 3.3. Hydrolysis Properties of PpRmMan134A

**Figure 5:**
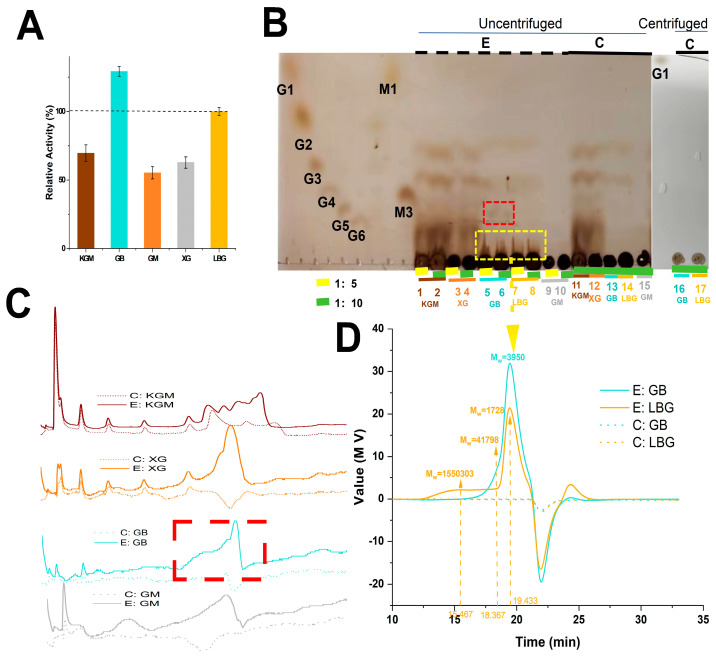
Analysis of hydrolysis properties of different substrates by *PpRm*Man134A. (**A**) Substrate specificity of *PpRm*Man134A. (**B**) Analysis of hydrolysis products from different substrates by *PpRm*Man134A. G1 (D-glucose), G2 (sucrose), G3 (maltotriose), G4 (maltotetraose), G5 (maltopentaose), G6 (maltohexaose), M1 (D-mannose), and M3 (mannotriose) are oligosaccharide standards; Lanes 1, 3, 5, 7, and 9 represent uncentrifuged reaction mixtures containing 0.2 mg *PpRm*Man134A in 0.1 mL 50 mM sodium citrate buffer (pH 5.5), which were incubated with 1 mL of various substrates at a concentration of 10 mg/mL; Lanes 2, 4, 6, 8, and 10 represent uncentrifuged reaction mixtures containing 0.4 mg *PpRm*Man134A in 0.2 mL 50 mM sodium citrate buffer (pH 5.5), which were incubated with 1 mL of various substrates at a concentration of 10 mg/mL; Lanes 11, 12, 13, 14, and 15 represent uncentrifuged reaction mixtures containing 0.1 mL of ddH_2_O and 1 mL of different substrates at a concentration of 10 mg/mL; Lanes 16 and 17 represent centrifuged reaction mixtures containing 0.1 mL ddH_2_O, which were mixed with 1 mL of either GB or LBG at a concentration of 10 mg/mL. (**C**) The oligosaccharide distribution produced by *PpRm*Man134A hydrolysis toward various substrates was analyzed. (**D**) The molecular weight distribution of GB and LBG hydrolyzed by *PpRm*Man134A was determined. Molecular weight (M_W_): unit, Da. In (**B**–**D**), *PpRm*Man134A reacted with different substrates for 20 h; E: *PpRm*Man134A with substrate; C: ddH_2_O with substrate (control).

The substrate specificity of *PpRm*Man134A was determined by employing various food-grade substrates. The enzyme displayed a specific activity of 100% towards locust bean gum (LBG), followed by konjac powder (KGM; 69.64%), guar gum (GB; 129.25%), glucomannan (GM; 55.24%), and xanthan gum (XG; 62.77%) (Figure 5A). This observation highlights the broad applicability of *PpRm*Man134A.

Figure 5B,C show that KGM and XG already contained different MOSs, making them unsuitable for studying the hydrolysates of *PpRm*Man134A. At the same time, GM itself also contained a small amount of MOS, and the products were composed of a variety of mixed MOSs (Figure 5C). Thus, GM hydrolysis products are irrelevant to this study.

Hydrolysis by *PpRm*Man134A of GB and LBG notably lowered the molecular weight (Figure 5B,D). Since GB and LBG contain precipitable MOS impurities and *PpRm*Man134A and LBG hydrolysis may yield small amounts of MOS (Figure 5B), no clear band was observed in the thin-layer chromatographic analysis of the hydrolysis products using LBG as the substrate. Therefore, HPLC analysis was necessary to detect MOS produced by the hydrolysis of *PpRm*Man134A toward LBG.

**Figure 6:**
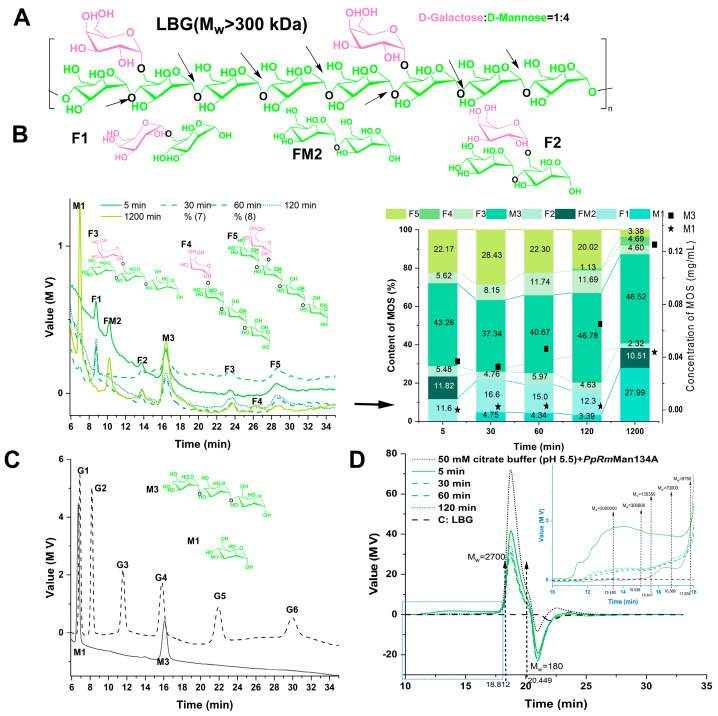
Product analysis of hydrolysis of locust bean gum by *PpRm*Man134A. C: ddH_2_O with substrate (control). (**A**) Possible structural formula of locust bean gum (LBG). The black arrows represent possible cleavage sites of *PpRm*Man134A. (**B**) Analysis of oligosaccharides from LBG hydrolysis by *PpRm*Man134A over time. F1, FM2, F2, F3, F4, and F5: unknown mannooligosaccharides. F1, 6^1^-α-D-galactosyl-α-D-mannose; M2, mannobiose; F2, 6^1^-α-D-galactosyl-β-D-mannobiose; F3, 6^1^-α-D-galactosyl-β-D-mannotriose; F4, 6^1^-α-D-galactosyl-α-D-mannotriose; F5, 6^1^-α-D-galactosyl-β-D-mannotetraose. (**C**) Standards for G1 (D-glucose), G2 (sucrose), G3 (maltotriose), G4 (maltotetraose), G5 (maltopentaose), G6 (maltohexaose), M1 (D-mannose), and M3 (mannotriose). (**D**) Molecular weight distribution of LBG products hydrolyzed by *PpRm*Man134A at different times. Molecular weight (M_W_): unit, Da.

Since mannotriose (M3) moved more slowly on silica gel than maltotriose (G3, Figure 5B), and LBG and GB had larger-molecular-weight products (Figure 5B,D), using mannotetraose, mannopentaose, and mannohexaose as standards would have affected calibration. Therefore, Figure 5B and Figure 6C also included D-glucose (G1), sucrose (G2), maltotriose (G3), maltotetraose (G4), maltopentaose (G5), maltohexaose (G6), and D-mannose (M1) as standards, arranged in the same order for both TCL and HPGFC methods (Figure 5B and Figure 6C).

The yield of MOS by *PpRm*Man134A hydrolysis using LBG as the substrate increased gradually with a prolongation of the reaction time, with mannotriose (M3) being the main product (37.34–46.78%) and a peak yield of 0.13 mg/mL (Figure 6B). The yield of D-mannose (M1) increased after hydrolysis for 1200 min, but the maximum yield was only 0.04 mg/mL (Figure 6B). In addition, previous experiments showed that *PpRm*Man134A dissolved in pure water does not react with LBG to produce oligosaccharides. Therefore, *PpRm*Man134A hydrolyzes LBG in a 50 mM pH 5.5 citric acid buffer. However, the mannooligosaccharides obtained contain significant citric acid and sodium citrate, causing an uneven baseline between 6 min and 14 min in the liquid phase diagram in Figure 6B.

Besides M1 and M3, six unverified MOS were detected. Based on LBG’s structure (Figure 6A) and amino columns in the HPLC method, F1, M2, F2, F3, F4, and F5 were speculated to be 6^1^-α-D-galactosyl-α-D-mannose, mannobiose, 6^1^-α-D-galactosyl-β-D-mannobiose, 6^1^-α-D-galactosyl-β-D-mannotriose, 6^1^-α-D-galactosyl-α-D-mannotriose, and 6^1^-α-D-galactosyl-β-D-mannotetraose (Figure 6B). Further research into the structure of these unknown components is planned in future studies.

F1, FM2, and F2 were positioned between M1 and M3; F1 and FM2 were between G2 and G3; and F2 was between G3 and G4 (Figure 6B). This suggests that F1 and FM2 are disaccharides and F2 is a trisaccharide. In the amino column, substances with higher polarities have longer elution times. Given that the keto group in the galactosyl group was less polar than the aldehyde group in the mannosyl group, F1, FM2, and F2 were likely 6^1^-α-D-galactosyl-α-D-mannose, mannobiose, and 6^1^-α-D-galactosyl-β-D-mannobiose, respectively. Given that the F3 and F5 peak positions were similar to G5 and G6, and M3 peak positions resembled G4 (Figure 6B), F3 and F5 were likely tetrasaccharides and pentasaccharides. Since the D-galactose to D-mannose ratio in LBG is 1:4 [41], and *PpRm*Man134A cleaves the β-1,4-glucoside bond in LBG, the hydrolysate of *PpRm*Man134A and LBG was unlikely to be mannotetraose or mannopentaose, but likely mannooligosaccharides with galactosyl. F4, positioned between F3 and F5, suggests that F3, F4, and F5 may correspond to 6^1^-α-D-galactosyl-β-D-mannotriose, 6^1^-α-D-galactosyl-α-D-mannotriose, and 6^1^-α-D-galactosyl-β-D-mannotetraose, respectively, based on the polarity of the galactosyl ketone group. During the first 30 min of LBG hydrolysis by *PpRm*Man134A, the levels of F1, F2, F4, and F5 increased (Figure 6B). However, from 30 min to 1200 min, their levels decreased (Figure 6B), suggesting they degrade over time. Specifically, between 60 min and 1200 min, the content of F5 dropped from 22.30% to 3.38%, while the content of F4 rose from 0% to 4.69%, indicating that F5 was converted to F4. Within 120–1200 min of hydrolysis, the contents of F3, F2 and F1 were greatly reduced, while the contents of FM2 and M1 were greatly increased. This suggests that F1 mainly hydrolyzed into M1, F3 into FM2, and F2 into both M1 and FM2.

Figure 6D also shows that as the hydrolysis time of *PpRm*Man134A increased from 5 to 60 min, the molecular weight of macromolecules (30 kDa–200 kDa) and MOS (180 Da–2700 Da) in LBG decreased. Additionally, extending the hydrolysis time to 2 h–20 h led to the degradation of LBG larger than 300 kDa into 200 kDa to 30 kDa macromolecules and MOSs with degrees of polymerization less than 6 (Figure 5C, Figure 6B and Figure 6D, respectively).

## 4. Conclusions

This study correlates ESPs in the fermentation supernatant with cell growth and death according to Equations (1)–(3). Schemes A8 and B3 produced *PpRm*Man134A with fewer extracellular proteins and higher enzyme activity than scheme B1, indicating that casein peptone should not be used as a nitrogen source in MBMMY medium. Enzyme activity increased modestly, ranging from 15.54% to 17.57%, between 48 and 72 h. This contrasts with the significant increase of 69.96% to 79.92% observed from 24 h to 48 h, which, according to Equation (2), was attributed to a higher WCW and decreasing energy efficiency for enzyme production. Shortening fermentation to 0 h–48 h in schemes C1–C9 and D1–D16 improved the energy utilization in the medium but decreased *PpRm*Man134A yield. To boost the yield, more *P. pastoris* was transferred to the MBMMY medium with an increased BMGY volume and glycerol. The better-evaluated conditions with 2% (*w*/*v*) methanol in MBMMY resulted in an enhanced enzyme activity and yield, albeit with increased ESPs, whereas lower methanol concentrations and 2% (*w*/*v*) peptone were the optimal conditions for *PpRm*Man134A production. So, culturing recombinant *P. pastoris* in 70 mL BMGY medium for 24 h at 30 °C, followed by 24 h at 30 °C in MBMMY medium with 0.91% (*w*/*v*) methanol, 0.56% (*w*/*v*) sorbitol, and 0.48% (*w*/*v*) mannitol, improved the production efficiency of *PpRm*Man134A. ESPs were reduced, and the fermentation time was cut by 72 h. Then, ultrafiltration and salt-out precipitation were used to purify the *PpRm*Man134A. *PpRm*Man134A retained 85% activity at 100 °C for 4 min, outperforming m*Rm*Man134A for high-temperature baking applications. LBG degradation by *PpRm*Man134A produced mannotriose with a peak yield of 0.13 mg/mL.

## Data Availability

The original contributions presented in this study are included in the article/Supplementary Materials, and further inquiries can be directed to the corresponding author.

## Supplementary Materials

The following supporting information can be downloaded at https://www.mdpi.com/article/10.3390/foods13203324/s1, Figure S1: Recombinant plasmid construction process; Figure S2: Comparison of m*Rm*Man134A and *PpRm*Man134A; Table S1: Better-evaluated conditions for enzymatic production of schemes A1–A9; Table S2: Better-evaluated conditions for enzymatic production of schemes B1–B5; Table S3: Better-evaluated conditions for enzymatic production of schemes C1–C10; Table S4: Better-evaluated conditions for enzymatic production of schemes D1–D16.
