# Supplementary material for: Purification and Characterization of β-Mannanase Derived from Rhizopus microsporus var. rhizopodiformis Expressed in Komagataella phaffii
Source: Foods. 2024 Oct 19;13(20):3324. doi: 10.3390/foods13203324 (PMC11507600; doi:10.3390/foods13203324)
Supplement: Supplementary file 1 [file foods-13-03324-s001.zip › foods-3228958-supplementary.pdf]

Supplementary figures

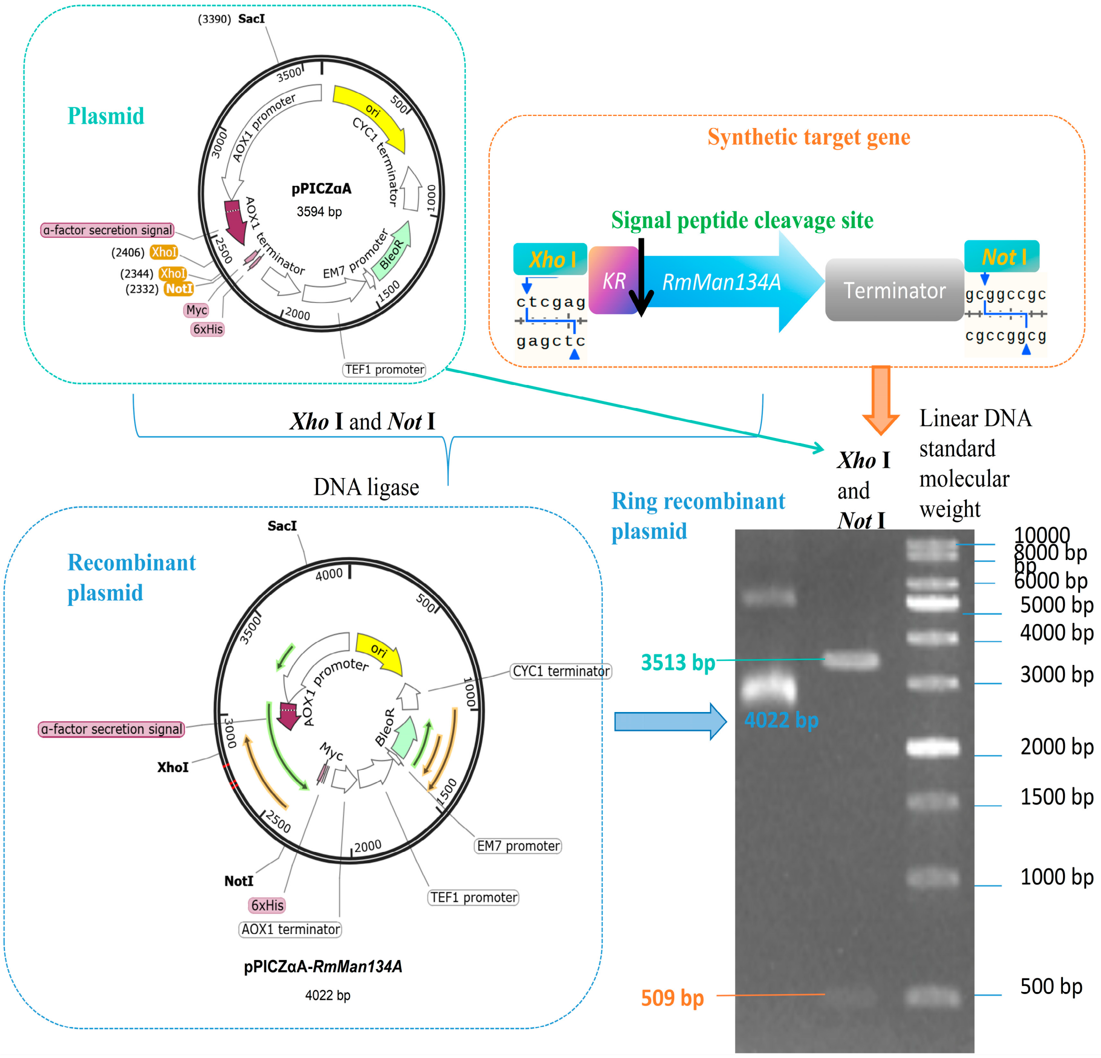

Figure S1. Recombinant plasmid construction process.

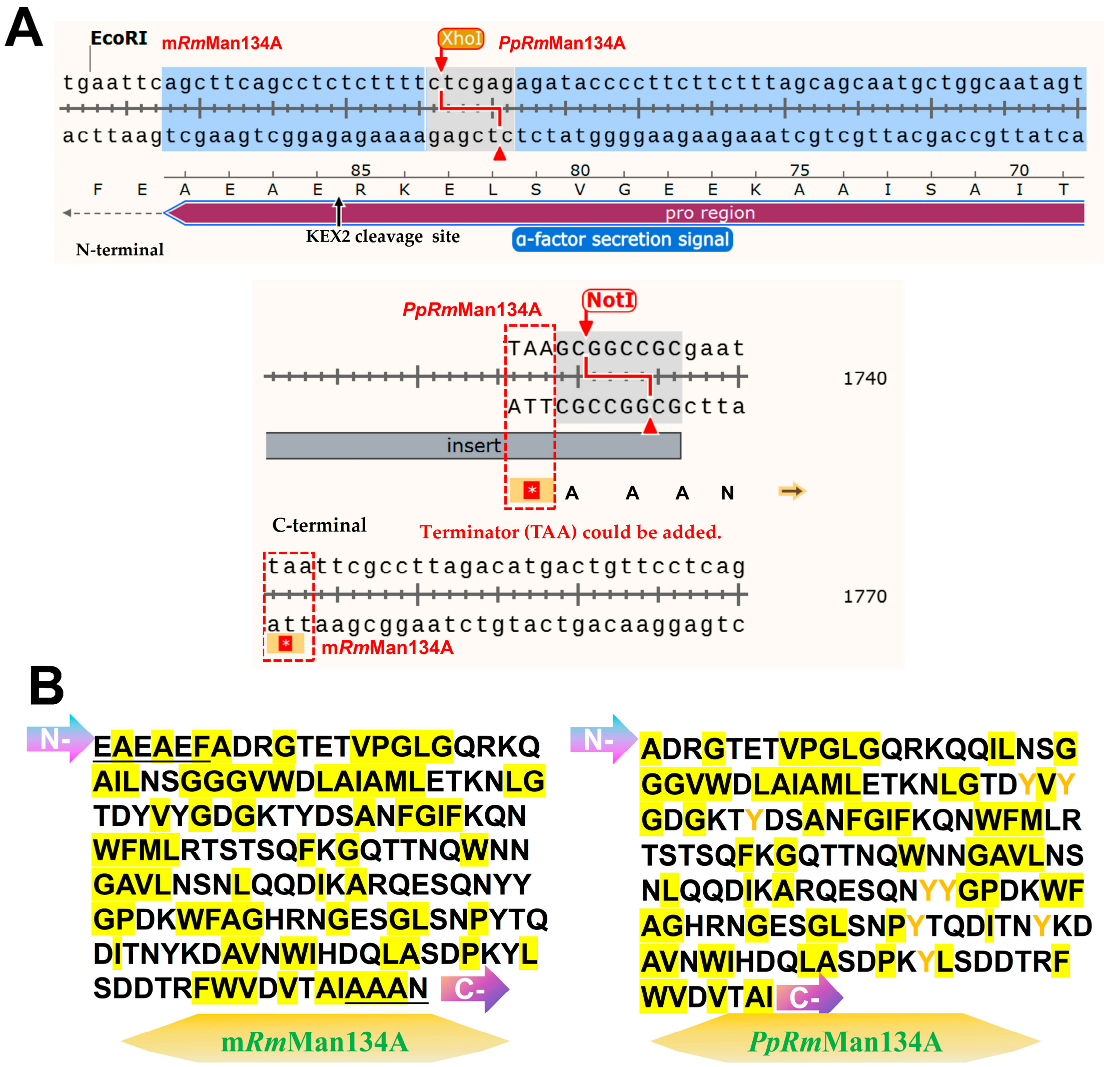

Figure S2. Comparison of m*Rm*Man134A and *PpRm*Man134A. (A) Variation in enzyme restriction sites used for constructing m*Rm*Man134A and *PpRm*Man134A. (B) Differences in amino acid sequences between m*Rm*Man134A and *PpRm*Man134A. N-: N-terminal; C-: C-terminal. Hydrophobic amino acids are highlighted in yellow. Underline represents excess amino acid sequences.

Supplementary tables

Note: Supplementary tables S1, S2, S3, and S4 detail the three evaluated conditions, which are categorized as low (-1), medium (0), and high (+1).

Table S1. Better-evaluated conditions for enzymatic production of schemes A1–A9.

50 mL BMGY medium (1 g peptone, 0.5 g yeast extract, 1 mL glycerin, 100 mM pH=6.37 potassium phosphate buffer)，30°C，200 rpm, 0 h–24 h															
100 mL MBMMY medium, 24 h–120 h, 30°C, 200 rpm															
Start and end time of culture				24 h–48 h			48 h–72 h			72 h–96 h			96 h–120 h		
Gro-up	Casein pepton	Pepton e	Yeast extract	Methanol	1 M sorbitol	1 M mannitol	Methanol	1 M sorbitol	1 M mannitol	Methanol	1 M sorbitol	1 M mannitol	Methanol	1 M sorbitol	1 M mannitol
1	_	2 g (+1)	1 g (0)	1000 μL (+1)	_	_	1000 μL (+1)	_	_	1000 μL (+1)	_	_	1000 μL (+1)	_	_
2	2 g (+1)	_	1 g (0)	1000 μL (+1)	_	_	1000 μL (+1)	_	_	1000 μL (+1)	_	_	1000 μL (+1)	_	_
3	_	2 g (+1)	1 g (0)	1110 μL (+1)	_	_	1110 μL (+1)	_	_	1110 μL (+1)	_	_	1110 μL (+1)	_	_
4	2 g (+1)	_	1 g (0)	1110 μL (+1)	_	_	1110 μL (+1)	_	_	1110 μL (+1)	_	_	1110 μL (+1)	_	_
5	_	2 g (+1)	1 g (0)	750 μL (0)	360 μL (+1)	_	1000 μL (0)	481 μL (+1)	_	875 μL (0)	420 μL (+1)	_	750 μL (0)	360 μL (+1)	_
6	2 g (+1)	_	1 g (0)	750 μL (0)	360 μL (+1)	_	1000 μL (0)	481 μL (+1)	_	875 μL (0)	420 μL (+1)	_	750 μL (0)	360 μL (+1)	_
7	_	2 g (+1)	1 g (0)	750 μL (0)	200 μL (0)	160 μL	1000 μL (0)	267 μL (0)	214 μL	875 μL (0)	233 μL (0)	187 μL	750 μL (0)	200 μL (0)	160 μL
8	2 g (+1)	_	1 g (0)	750 μL (0)	200 μL (0)	160 μL	1000 μL (0)	267 μL (0)	214 μL	875 μL (0)	233 μL (0)	187 μL	750 μL (0)	200 μL (0)	160 μL
9	_	2 g (+1)	2 g (+1)	750 μL (0)	200 μL (0)	160 μL	1000 μL (0)	267 μL (0)	214 μL	875 μL (0)	233 μL (0)	187 μL	750 μL (0)	200 μL (0)	160 μL

Table S2. Better-evaluated conditions for enzymatic production of schemes B1 – B5.

50 mL BMGY medium (1 g peptone, 0.5 g yeast extract, 1 mL glycerin, 100 mM pH=6.37 potassium phosphate buffer)，30°C，200 rpm, 0 h–24 h														
100 mL MBMMY medium, 24 h–120 h, 200 rpm														
Start and end time of culture			24 h–48 h, 30°C			48 h–72 h, 29°C			72 h–96 h, 28°C			96 h–120 h, 27.2°C		
Gro-up	Casein peptone	Yeast extract	Methanol	1 M sorbitol	1 M mannitol	Methanol	1 M sorbitol	1 M mannitol	Methanol	1 M sorbitol	1 M mannitol	Methanol	1 M sorbitol	1 M mannitol
1	2 g (+1)	1 g (0)	1110 μL (+1)	_	_	1110 μL (+1)	_	_	1110 μL (+1)	_	_	1110 μL (+1)	_	_
2	2 g (+1)	1 g (0)	750 μL (0)	360 μL (+1)	_	1000 μL (0)	481 μL (+1)	_	875 μL (0)	420 μL (+1)	_	750 μL (0)	360 μL (+1)	_
3	2 g (+1)	1 g (0)	750 μL (0)	200 μL (0)	160 μL	1000 μL (0)	267 μL (0)	214 μL	875 μL (0)	233 μL (0)	187 μL	750 μL (0)	200 μL (0)	160 μL
4	1 g (0)	1 g (0)	750 μL (0)	200 μL (0)	160 μL	1000 μL (0)	267 μL (0)	214 μL	875 μL (0)	233 μL (0)	187 μL	750 μL (0)	200 μL (0)	160 μL
5	0.5 g (-1)	0.5 g (-1)	750 μL (0)	200 μL (0)	160 μL	1000 μL (0)	267 μL (0)	214 μL	875 μL (0)	233 μL (0)	187 μL	750 μL (0)	200 μL (0)	160 μL

Table S3. Better-evaluated conditions for enzymatic production of schemes C1﹣C10.

70 mL BMGY medium (1.4 g peptone，0.7 g yeast extract，2 mL glycerin, pH=6.37 potassium phosphate buffer) ，30°C，200 rpm,0 h–24 h

100 mL MBMMY medium，200 rpm, 30°C, 24 h–48 h

Group	Peptone	Yeast extract	Buffer concentration	Methanol	1 M sorbitol	1 M mannitol
1	1 g (0)	1 g (0)	100 mM (+1)	2550 μL (+1)	680 μL	—
2	2 g (+1)	1 g (0)	100 mM (+1)	2550 μL (+1)	680 μL	—
3	2 g (+1)	1 g (0)	100 mM (+1)	2550 μL (+1)	680 μL	585 μL
5	1 g (0)	1 g (0)	100 mM (+1)	2550 μL (+1)	680 μL	585 μL
7	2 g (+1)	1 g (0)	80 mM (0)	2550 μL (+1)	680 μL	585 μL
9	1 g (0)	1 g (0)	80 mM (0)	2550 μL (+1)	680 μL	585 μL

70 mL BMGY medium (1.4 g casein peptone，0.7 g yeast extract，2 mL glycerin, pH=6.37 potassium phosphate buffer) ，30°C，200 rpm, 0 h–24 h

100 mL MBMMY medium，200 rpm, 30°C, 24-48 h

Group	Peptone	Yeast extract	Buffer concentration	Methanol	1 M sorbitol	1 M mannitol
4	2 g (+1)	1 g (0)	100 mM (+1)	2550 μL (+1)	680 μL	585 μL
6	1 g (0)	1 g (0)	100 mM (+1)	2550 μL (+1)	680 μL	585 μL
8	2 g (+1)	1 g (0)	80 mM (0)	2550 μL (+1)	680 μL	585 μL
10	1 g (0)	1 g (0)	80 mM (0)	2550 μL (+1)	680 μL	585 μL

Table S4. Better-evaluated conditions for enzymatic production of schemes D1–D16.

70 mL BMGY medium (1.4 g casein peptone，0.7 g yeast extract，2mL glycerin, 100 mM pH=6.37 potassium phosphate buffer)，30°C，200 rpm, 0 h–24 h					
100 mL MBMMY medium，200 rpm, 30°C, 24 h–48 h					
Group	Peptone	Yeast extract	Methanol	1 M sorbitol	1 M mannitol
1	2 g (+1)	1 g (0)	1950 μL (0)	680 μL	585 μL
2	1 g (0)	1 g (0)	1950 μL (0)	680 μL	585 μL
3	2 g (+1)	1 g (0)	2150 μL (+1)	680 μL	585 μL
4	1 g (0)	1 g (0)	2150 μL (+1)	680 μL	585 μL
5	2 g (+1)	1 g (0)	2350 μL (+1)	680 μL	585 μL
6	1 g (0)	1 g (0)	2350 μL (+1)	680 μL	585 μL
7	2 g (+1)	1 g (0)	2550 μL (+1)	680 μL	585 μL
8	1 g (0)	1 g (0)	2550 μL (+1)	680 μL	585 μL
9	2 g (+1)	1 g (0)	1150 μL (-1)	680 μL	585 μL
10	1 g (0)	1 g (0)	1150 μL (-1)	680 μL	585 μL
11	2 g (+1)	1 g (0)	1350 μL (-1)	680 μL	585 μL
12	1 g (0)	1 g (0)	1350 μL (-1)	680 μL	585 μL
13	2 g (+1)	1 g (0)	1550 μL (-1)	680 μL	585 μL
14	1 g (0)	1 g (0)	1550 μL (-1)	680 μL	585 μL
15	2 g (+1)	1 g (0)	1750 μL (0)	680 μL	585 μL
16	1 g (0)	1 g (0)	1750 μL (0)	680 μL	585 μL
